# Hepatocellular carcinoma outcomes and potential implications for surveillance in elderly patients

**DOI:** 10.1038/s41598-024-66253-0

**Published:** 2024-07-04

**Authors:** Aryoung Kim, Goeun Park, Myung Ji Goh, Byeong Geun Song, Wonseok Kang, Geum-Youn Gwak, Yong-Han Paik, Moon Seok Choi, Joon Hyeok Lee, Dong Hyun Sinn

**Affiliations:** 1 Department of Medicine, Samsung Medical Center, Sungkyunkwan University School of Medicine, 81 Irwon-ro, Gangnam-gu, 06351 Seoul, South Korea; 2 Department of Internal Medicine, Inje University Ilsan Paik Hospital, Goyang, Korea; 3 Research Institute for Future Medicine, Biomedical Statistics Center, Samsung Medical Center, Seoul, Korea; 4 Department of Health Sciences and Technology, Samsung Advanced Institute for Health Sciences and Technology, Sungkyunkwan University, Seoul, Korea

**Keywords:** Hepatocellular carcinoma, Surveillance, Elderly, Hepatology, Cancer screening

## Abstract

International liver societies recommend hepatocellular carcinoma (HCC) surveillance for those at high-risk of developing HCC. While previous studies have shown the benefits of surveillance for middle-aged patients, but its necessity for elderly patients is unclear. This study aimed to assess the benefits of HCC surveillance in the elderly by comparing diagnosis mode of HCC. Consecutive, elderly patients aged 75 years or older who were newly diagnosed with HCC were screened at our institution between January 2009 and December 2021. Patients were grouped into those who were diagnosed with HCC during surveillance (*n* = 235, surveillance group) and those who were diagnosed with HCC due to symptoms (*n* = 184, symptomatic group). The study outcome was overall survival. It was compared in the overall cohort and a propensity score (PS)-matched cohort. Early-stage diagnosis was more frequent in the surveillance group than in the symptomatic group (mUICC stage I/II: 72.3% vs. 39.1%, *p* < 0.001). The overall survival rate was better in the surveillance group than in the symptomatic group (median 4.4 vs. 2.1 years, log-rank *p* < 0.001). In multivariable-adjusted models, the hazard ratio (HR) of mortality of the surveillance group compared to the symptomatic group was 0.64 (95% confidence interval (CI): 0.47–0.87). However, further adjustment for the tumor stage markedly attenuated this association, which was no longer statistically significant (adjusted HR = 0.75; 95% CI: 0.54–1.02). In the PS-matched cohort analysis, outcomes were similar when the PS matching variables included the tumor stage. In contrast, when PS matching variables did not include the tumor stage, outcomes were better for the surveillance group. The surveillance group of elderly patients showed better survival than the symptomatic group, which was largely explained by earlier tumor stage at diagnosis. This suggests that the overall outcome of elderly HCC patients could be improved by increasing surveillance-detected cases compared to symptom-driven cases.

## Introduction

Hepatocellular carcinoma (HCC) is the third leading cause of cancer-related death worldwide^1^, with increasing incidence and mortality in both Western and Asian countries^2,3^. The prognosis of patients presenting with advanced-stage HCC is disastrous. Curative treatment options are only available for patients diagnosed with HCC at an early stage^4^. Several studies have shown that HCC surveillance was associated with improved early detection, receipt of curative treatment, and survival of patients at a high risk of developing HCC^5,6^. Therefore, HCC surveillance is recommended by international liver societies for high-risk patients^5,7,8^.

However, surveillance is not always beneficial. Cancer surveillance can lead to physical complications, psychological distress, and financial burden^9^. The overall value of HCC surveillance must balance the benefit of reducing cancer mortality against potential harms. Negative effects of screening, such as overdiagnosis of clinically insignificant disorders, complications from diagnostic procedures, and distress after false positive test results, might be more harmful to elderly populations. Risks and benefits of surveillance for breast, prostate, colorectal, lung, and cervical cancers in elderly populations have been evaluated^10^. However, information on HCC surveillance in the elderly is limited^11^. To date, two randomized controlled studies have analyzed the effect of HCC surveillance on mortality. These studies were conducted in middle-aged individuals with chronic hepatitis B infection or a history of chronic hepatitis. Patients who underwent surveillance were more likely to have early-stage HCC detected and undergo curative therapy, indicating the necessity of surveillance in high-risk middle-aged patients^12,13^. However, to the best of our knowledge, no randomized controlled trial has assessed the risk and benefit of HCC surveillance in an elderly population. Although randomized controlled trials are required to provide a high level of evidence, such trials require huge medical resources. From this perspective, cohort studies can give valuable insight into these issues. Medical technology and healthcare services are evolving. In the meantime, the general population and HCC patients are aging^14^. Hence, this study aimed to assess whether surveillance contributes to early detection and improves survival in elderly HCC patients by categorizing patients according to the mode of HCC diagnosis.

## Methods

### Study design and population

**Figure 1:**
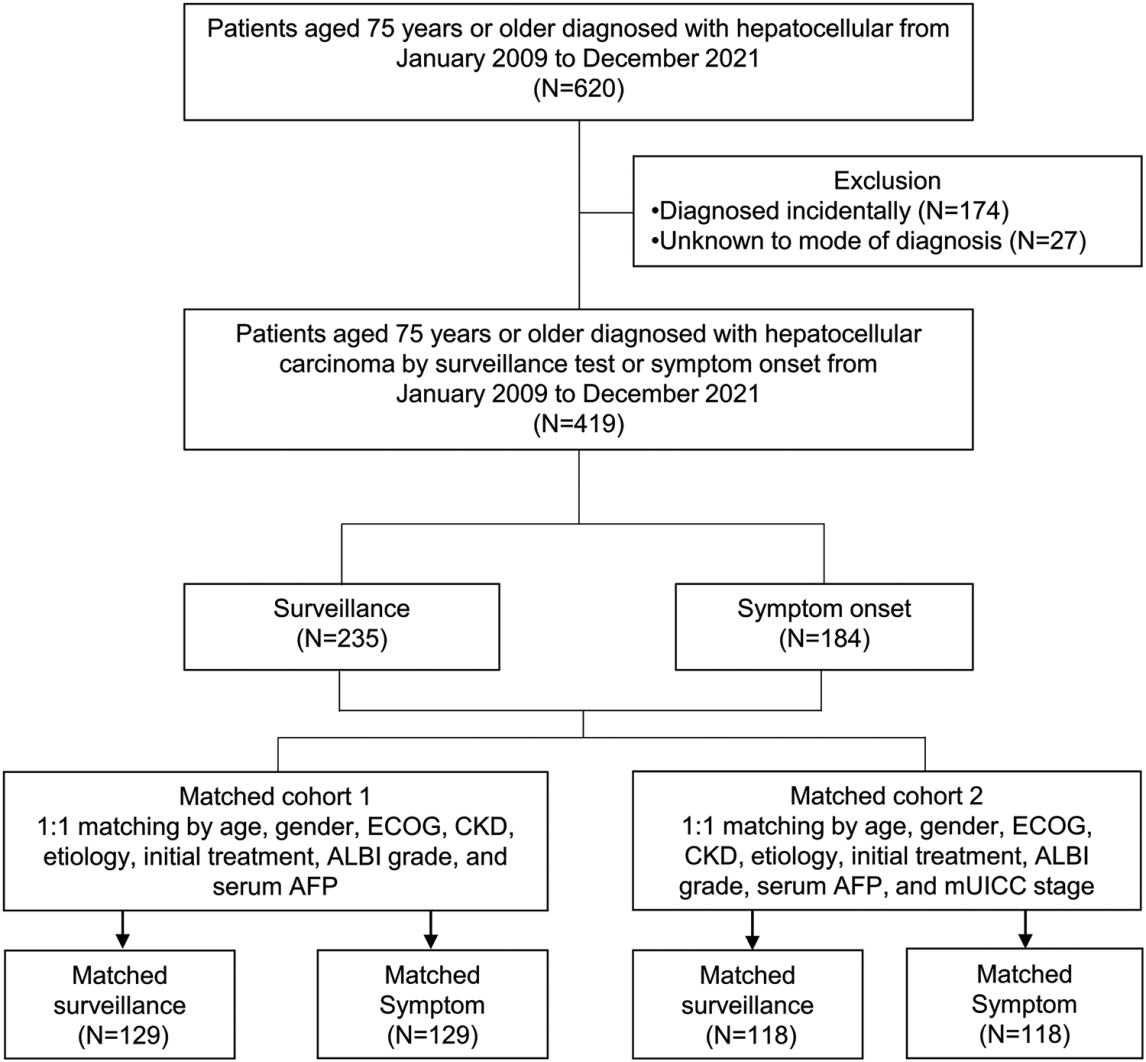
Study population diagram. ECOG, Eastern Cooperative Oncology Group; CKD, chronic kidney disease; ALBI, albumin-bilirubin; AFP, α-fetoprotein; mUICC, modified Union for International Cancer Control.

This was a single-center retrospective cohort study. Through an electronic medical records (EMR) review, we screened on newly diagnosed HCC patients aged 75 years or older between January 2009 and December 2021. Since our primary goal was to assess the survival rate of HCC patients based on surveillance, we initially screened individuals who didn’t have other malignancies that could impact their survival (*n* = 620) (Fig. 1). Then, we reviewed each patient’s mode of HCC diagnosis using EMR. Our institution has been mandated to record the mode of diagnosis of patients in the EMR since 2009. The diagnosis mode was categorized into surveillance, incidental, and symptomatic. HCC surveillance cases were defined as routine screening with ultrasonography (US) with or without blood α-fetoprotein [AFP] measures 6 months prior to the first identification of HCC in high-risk individuals with chronic liver disorders such viral hepatitis or cirrhosis. For some patients where US was inadequate, computed tomography or magnetic resonance imaging was utilized. Incidental cases were defined as asymptomatic cases, where diagnostic tests for HCC were not triggered by HCC screening tests (e.g., a health check-up or evaluation for other medical conditions). Symptomatic cases were defined when diagnostic tests for HCC were triggered by symptoms. To assess the usefulness of the surveillance, which is regularly performed with the goal of diagnosing before symptoms appear, we excluded patients who were diagnosed incidentally (*n* = 174) or by an unknown mode (*n* = 27). Finally, 419 patients were analyzed in this study (Fig. 1).

### Study outcomes and variables

The study’s endpoint was overall survival (OS), defined as the time from the diagnosis of HCC to patient’s death or the date of the last follow-up. When a patient died, the date of death was recorded in the hospital’s EMR, therefore this record was used to determine the date of death, and for surviving patients, the date of last visit was censored. The mode of HCC diagnosis was either by surveillance or symptoms. The following variables were collected as potential confounders or mediators by reviewing the EMR of each patient: age at HCC diagnosis, gender, Eastern Cooperative Oncology Group (ECOG) performance score, underlying comorbidity (diabetes mellitus, hypertension, or chronic kidney disease), etiology of HCC, Child–Pugh score, albumin-bilirubin (ALBI) grade, modified Union for International Cancer Control (mUICC) stage, initial treatment, total bilirubin, aspartate aminotransferase (AST), alanine aminotransferase (ALT), creatinine, estimated glomerular filtration rate (eGFR), α-fetoprotein (AFP), and prothrombin induced by vitamin K absence II (PIVKA II) levels. mUICC stage I or stage II was considered as early stage HCC^15^.

### Statistical analysis

The baseline characteristics were compared using Mann–Whitney U tests for continuous variables and the χ^2^ test or Fisher’s exact test for categorical variables. Survival was estimated using the Kaplan–Meier method and compared by the log-rank test to analyze differences in OS between groups. For risk analysis, we calculated hazard ratios (HRs) with 95% confidence intervals (CIs) for all-cause mortality using Cox proportional hazard regression models. We used 3 models with increasing degrees of adjustment to account for potential confounding factors at baseline. Model 1 was adjusted for age (years), gender (male vs. female), ECOG score (0 vs. 1–4), chronic kidney disease (eGFR ≥ 60 vs. < 60 mL/min/1.73 m^2^), and etiology of HCC (viral vs. non-viral). Model 2 was further adjusted for initial treatment (received treatment vs. no treatment), ALBI grade (1 vs. 2–3), and serum AFP levels (/log_10_ ug/mL). Model 3 was further adjusted for the mUICC stage (1–2 vs. 3–4). We performed subgroup analysis to evaluate whether associations between OS and diagnosis mode differed in prespecified subgroups (age 75–79 years or ≥ 80 years).

Next, we generated two propensity score-matched cohorts to investigate whether the variance in survival rates between the surveillance and symptomatic groups could be attributed to tumor stage. The following variables were selected for the first propensity score cohort and used to match surveillance and symptomatic groups: age, gender, ECOG score, chronic kidney disease, etiology of HCC, initial treatment, ALBI grade, and serum AFP levels. The tumor stage was not included in the first propensity score-matched variables. Thus, the first propensity score matching analysis was performed with only tumor stage differing between the groups. Subsequently, mUICC stage was used to generate the second propensity score-matched cohort, along with the variables used in generating the first propensity score-matched cohort. By comparing the first and second PS matching analyses, focusing solely on differences in tumor stage, we sought to ascertain the impact of tumor stage on the observed difference in survival rates. Propensity matching was done at a 1:1 ratio using nearest-neighbor greedy matching without replacement of the propensity score (caliper: 0.20) to select two groups of patients with balanced characteristics. Standardized mean differences were used to balance baseline scores. Most values were ≤ 0.15 after matching. After matching, the baseline characteristics were compared using the Wilcoxon signed-rank test for continuous variables and the marginal logistic regression model with generalized estimating equation (GEE) for categorical variables. Survivals in the propensity score-matched set were estimated using the Kaplan–Meier method. HRs with 95% CIs for all-cause mortality were calculated using marginal Cox regression models. All analyses were performed using SAS version 9.4 (SAS Institute, Cary, NC, USA). A two-tailed *p* < 0.05 was considered significant.

### Ethics approval statement

This study was conducted after approval was obtained from the Institutional Review Board of Samsung Medical Center (approval no. 2023-09-069) and was conducted in accordance with the Declaration of Helsinki. The requirement to obtain informed consent was waived by Institutional Review Board of Samsung Medical Center as we used only de-identified data routinely collected during hospital visits.

## Results

### Baseline characteristics

The baseline characteristics of the study patients are shown in Table 1. There was no difference in age, comorbidity (diabetes mellitus, hypertension, and CKD), or underlying liver function (Child–Pugh score and ALBI grade) between the surveillance and the symptomatic groups. Compared to patients with symptoms, those diagnosed by surveillance were more likely to be female (female 41.7% vs. 30.4%, *p* = 0.018), have better ECOG performance (ECOG 0 78.7% vs. 60.9%, *p* < 0.001), more viral etiology (HBV 32.8% vs. 15.2%; HCV 30.6% vs. 14.1%, *p* < 0.001), more likely to be diagnosed with mUICC stage 1 or 2 (72.3% vs. 39.1%, *p* < 0.001), and have lower serum tumor marker levels (median AFP 9.2 vs. 35.2 μg/mL, *p* < 0.001; median PIVKA II 33 vs.1725 mAU/mL, *p* < 0.001). Among patients with viral hepatitis, 44.3% (66/149) took antiviral medications in the surveillance group, compared to 35.2% (19/54) in the symptomatic group (*p* = 0.32).

**Table 1:** Baseline characteristics of the included individuals.

	Overall cohort		
	Surveillance (*n* = 235)	Sx (*n* = 184)	*p*-value
Age (years)			0.71
75–80	166 (70.6%)	133 (72.3%)	
≥ 80	69 (29.4%)	51 (27.7%)	
Gender (Male)	137 (58.3%)	128 (69.6%)	0.018
ECOG			< 0.001
0	185 (78.7%)	112 (60.9%)	
≥ 1	50 (21.3%)	72 (39.1%)	
Diabetes mellitus	89 (37.9%)	76 (41.3%)	0.48
Hypertension	145 (61.7%)	106 (57.6%)	0.40
CKD	75 (31.9%)	59 (32.1%)	0.97
Etiology			< 0.001
HBV^a^	77 (32.8%)	28 (15.2%)	
HCV	72 (30.6%)	26 (14.1%)	
Alcohol	31 (13.2%)	38 (20.7%)	
Others^b^	55 (23.4%)	92 (50.0%)	
Child–Pugh class			0.89
A	201 (85.5%)	155 (84.2%)	
B	31 (13.2%)	27 (14.7%)	
C	3 (1.3%)	2 (1.1%)	
ALBI grade			0.58
1	152 (64.7%)	110 (59.8%)	
2	78 (33.2%)	70 (38.0%)	
3	5 (2.1%)	4 (2.2%)	
mUICC stage			< 0.001
I	72 (30.6%)	8 (4.3%)	
II	98 (41.7%)	64 (34.8%)	
III	50 (21.3%)	45 (24.5%)	
IV-A	10 (4.3%)	31 (16.8%)	
IV-B	5 (2.1%)	36 (19.6%)	
Initial treatment (Yes)	218 (92.8%)	167 (90.8%)	0.46
Resection	66 (28.1%)	9 (4.9%)	
Local ablative therapy	44 (18.7%)	45 (24.5%)	
Transarterial therapy	83 (35.3%)	69 (37.5%)	
Systemic therapy	7 (3.0%)	26 (14.1%)	
Combination of two locoregional therapies^c^	14 (6.0%)	5 (2.7%)	
Others^d^	4 (1.7%)	13 (7.1%)	
Lab			
Total bilirubin (mg/dL)	0.8 (0.5–1.1)	0.7 (0.5–1.0)	0.05
Aspartate transaminase (U/L)	36 (27–54)	45 (30–69)	< 0.001
Alanine transaminase (U/L)	23 (17–33)	28 (20–44)	< 0.001
Creatinine (mg/dL)	0.89 (0.74–1.05)	0.91 (0.76–1.1)	0.28
eGFR (mL/min/1.73 m^2^)	72 (56–90)	69 (55–90)	0.63
α-fetoprotein (ug/mL)^e^	9.2 (3.6–44.0)	35.2 (4.3–719.3)	< 0.001
PIVKA -II (mAU/mL)^f^	33 (21–228)	1725 (78–13,421)	< 0.001

Sx, symptom; ECOG, Eastern Cooperative Oncology Group; CKD, chronic kidney disease; HBV, hepatitis B virus; HCV, hepatitis C virus; ALBI, albumin-bilirubin; mUICC, modified Union for International Cancer Control; eGFR, estimated glomerular filtration rate; AFP, α-fetoprotein; PIVKA II, prothrombin induced by vitamin K absence II.

^a^Six patients with both HBV and HCV as the cause of HCC were included in HBV.

^b^Others included non-hepatitis B-non hepatitis C (NBNC), autoimmune hepatitis, cryptogenic.

^c^transarterial plus ablation (n = 10), transarteral plus radiation therapy (n = 7), or transarteral plus proton therapy (n = 2).

^d^Others included radiation therapy (n = 11) or intrahepatic proton therapy (n = 6).

^e^A value was missing in 2 patients.

^f^A value was missing in 38 patients.

### Diagnosis mode and OS

**Figure 2:**
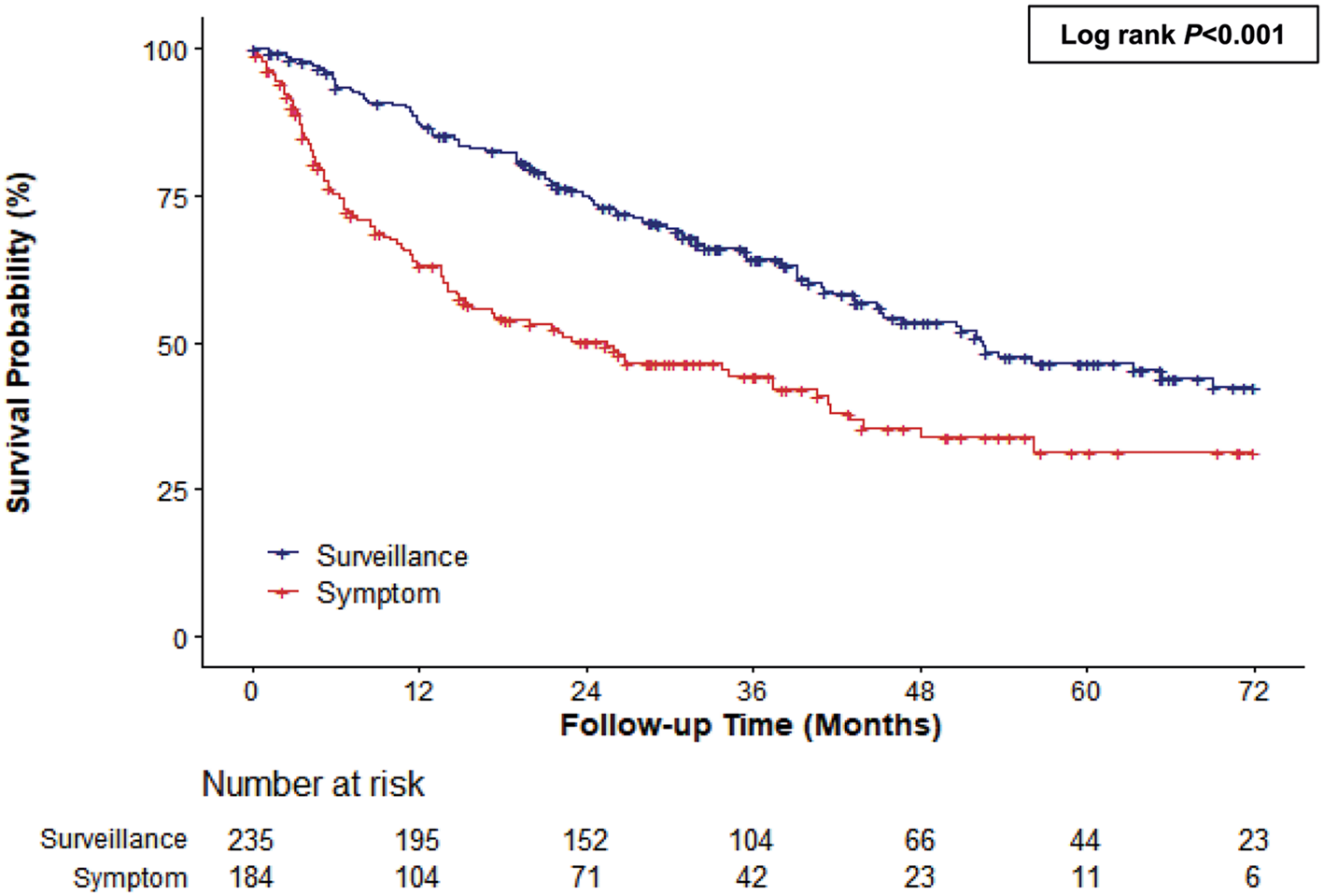
Kaplan–Meier estimates of survival in elderly patients according to diagnosis mode (over 75 years old).

The median survival was 3.5 years (range: 0.0–6.0 years). OS was better in the surveillance group than in the symptomatic group (median 4.4 vs. 2.1 years, log-rank *p* < 0.001) (Fig. 2). The diagnosis mode was associated with mortality risk. The mortality risk was lower in the surveillance group than in the symptomatic group (HR = 0.51, 95% CI: 0.39–0.68). In the multivariable-adjusted model, the adjusted HR for mortality, after adjusting for age, gender, ECOG, etiology, and CKD was 0.56 (95% CI: 0.41–0.77). This association remained significant after further adjustment for cancer treatment, ALBI grade, and AFP level (aHR = 0.64, 95% CI: 0.47–0.87). However, when we further adjusted for the mUICC stage, the association between diagnosis mode and mortality was markedly attenuated and was no longer statistically significant (HR = 0.75, 95% CI: 0.54–1.03) (Table 2).

**Table 2:** Cox-regression for diagnosis mode and mortality in elderly patients (over 75 years old).

	Univariable Cox regression		Multivariable Cox Regression Model 1		Multivariable Cox Regression Model 2		Multivariable Cox Regression Model 3	
	HR (95% CI)	*p*-value	aHR (95% CI)	*p*-value	aHR (95% CI)	*p*-value	aHR (95% CI)	*p*-value
Mode of diagnosis								
Symptom onset	Reference		Reference		Reference		Reference	
Surveillance	0.51 (0.39–0.68)	< 0.001	0.56 (0.41–0.77)	< 0.001	0.64 (0.47–0.87)	0.004	0.75 (0.54–1.03)	0.07
Age (years)	1.06 (1.02–1.11)	0.007	1.05 (1.01–1.10)	0.025	1.05 (1.01–1.10)	0.017	1.04 (1.00–1.09)	0.06
Gender (Male)	1.13 (0.85–1.50)	0.41	0.91 (0.66–1.26)	0.58	1.04 (0.74–1.44)	0.84	0.93 (0.66–1.30)	0.66
ECOG								
0	Reference		Reference		Reference		Reference	
≥ 1	1.28 (0.95–1.72)	0.11	1.13 (0.83–1.54)	0.45	0.80 (0.59–1.11)	0.18	0.83 (0.60–1.14)	0.24
CKD (Yes)	1.40 (1.05–1.86)	0.021	1.31 (0.96–1.78)	0.09	1.24 (0.90–1.71)	0.19	1.35 (0.98–1.87)	0.07
Etiology								
Viral	Reference		Reference		Reference		Reference	
Non-viral	1.49 (1.13–1.97)	0.005	1.19 (0.87–1.62)	0.28	1.55 (1.14–2.10)	0.005	1.65 (1.22–2.23)	0.001
Initial treatment (Yes)	0.23 (0.15–0.33)	< 0.001			0.32 (0.21–0.48)	< 0.001	0.35 (0.23–0.52)	< 0.001
ALBI grade								
1	Reference				Reference		Reference	
2–3	3.25 (2.45–4.31)	< 0.001			3.42 (2.53–4.63)	< 0.001	3.18 (2.35–4.31)	< 0.001
α-fetoprotein (log10 ug/mL)	1.41 (1.28–1.55)	< 0.001			1.44 (1.30–1.59)	< 0.001	1.35 (1.22–1.50)	< 0.001
mUICC stage								
I–II	Reference						Reference	
III–IV	2.76 (2.08–3.66)	< 0.001					1.86 (1.35–2.58)	< 0.001

HR, hazard ratio; aHR, adjusted hazard ratio; ECOG, Eastern Cooperative Oncology Group; CKD, chronic kidney disease; HCC, hepatocellular carcinoma; ALBI, albumin-bilirubin; AFP, α-fetoprotein; mUICC, modified Union for International Cancer Control.

**Model 1:** adjusted for age (years), gender (male vs. female), ECOG (0 vs. 1–4), CKD (eGFR ≥ 60 vs. < 60 mL/min/1.73 m^2^), and etiology of HCC (viral vs. non-viral).

**Model 2:** adjusted for age (years), gender (male vs. female), ECOG (0 vs. 1–4), CKD (eGFR ≥ 60 vs. < 60 mL/min/1.73 m^2^), etiology of HCC (viral vs. non-viral), initial treatment (yes vs. no), ALBI grade (1 vs. 2–3), and serum AFP (/log_10_ ug/mL).

**Model 3:** adjusted for age (years), gender (male vs. female), ECOG (0 vs. 1–4), CKD (eGFR ≥ 60 vs. < 60 mL/min/1.73 m^2^), etiology of HCC (viral vs. non-viral), initial treatment (yes vs. no), ALBI grade (1 vs. 2–3), serum AFP (/log_10_ ug/mL), and mUICC stage (1–2 vs. 3–4).

### Subgroup analysis by age

**Figure 3:**
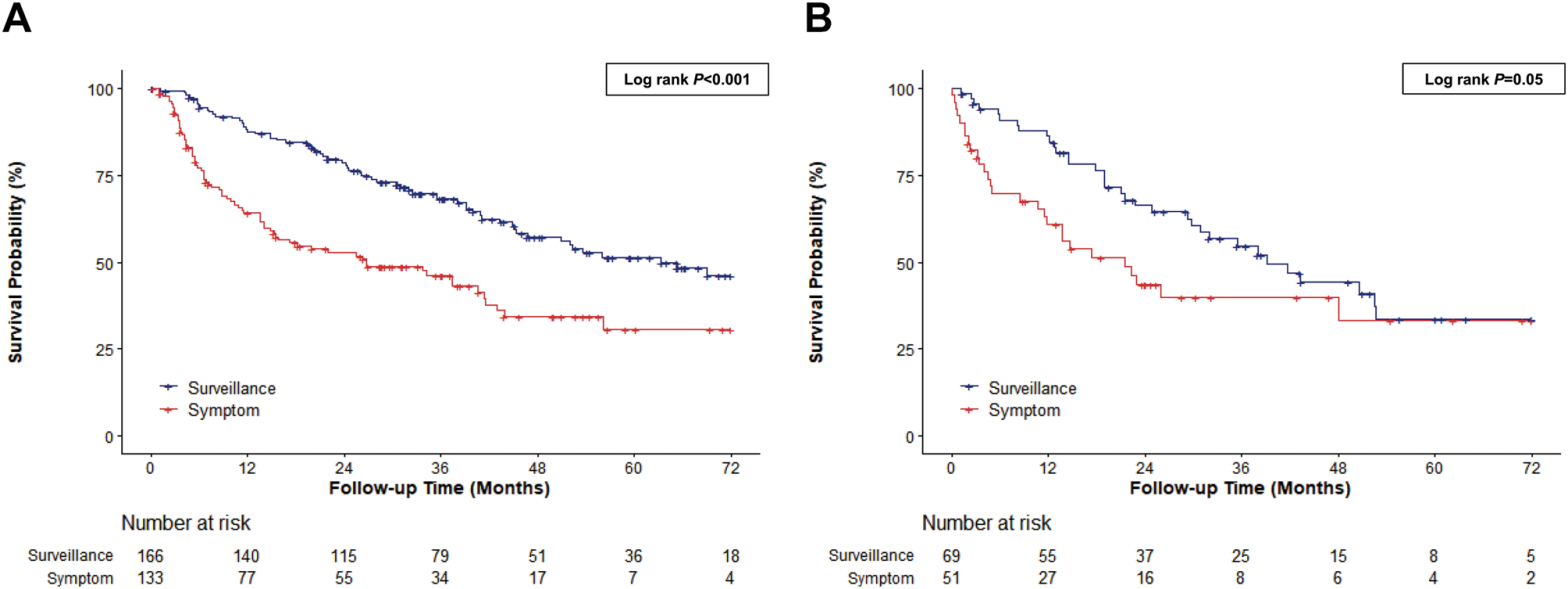
Kaplan–Meier estimates of survival in (**A**) 75–79 years, (**B**) over 80 years patients according to diagnosis mode.

The median survival of patients aged 75–79 years was longer in the surveillance group than in the symptomatic group (median 5.3 vs. 2.2 years, log-rank *p* < 0.001) (Fig. 3A). The mortality risk was lower in the surveillance group than in the symptomatic group in univariable analysis, but not in the fully-adjusted model that included tumor stage (Table 3). The median survival of patients aged 80 years or older was better in the surveillance group than in the symptomatic group (median 3.3 vs. 1.8 years, log-rank *p* = 0.05) (Fig. 3B). The diagnosis mode was marginally associated with mortality in univariable analysis. However, this association disappeared in the multivariable-adjusted model that included the mUICC stage (Table 3).

**Table 3:** Cox regression analysis of diagnosis by surveillance and mortality stratified by age.

Age of diagnosis^a^	Univariable Cox regression		Fully adjusted multivariable Cox regression model 3	
	HR (95% CI)	*p*-value	aHR (95% CI)	*p*-value
75–79 years (*n* = 299)				
Mode of diagnosis				
Symptom onset	Reference		Reference	
Surveillance	0.47 (0.33–0.66)	< 0.001	0.88 (0.58–1.32)	0.53
Age (years)	1.03 (0.91–1.18)	0.61	1.07 (0.94–1.23)	0.30
Gender (Male)	1.35 (0.94–1.93)	0.11	0.95 (0.63–1.45)	0.82
ECOG				
0	Reference		Reference	
≥ 1	1.25 (0.86–1.80)	0.24	0.74 (0.50–1.11)	0.14
CKD (Yes)	1.55 (1.10–2.19)	0.012	1.51 (1.02–2.24)	0.038
Etiology				
Viral	Reference		Reference	
Non-viral	1.70 (1.21–2.39)	0.002	1.79 (1.22–2.61)	0.003
Initial treatment (Yes)	0.27 (0.16–0.45)	< 0.001	0.43 (0.24–0.76)	0.004
ALBI grade				
1	Reference		Reference	
2–3	3.35 (2.38–4.71)	< 0.001	3.72 (2.55–5.42)	< 0.001
α-fetoprotein (log10 ug/mL)	1.50 (1.33–1.68)	< 0.001	1.50 (1.31–1.72)	< 0.001
mUICC stage				
I–II	Reference		Reference	
III–IV	2.75 (1.97–86)	< 0.001	1.95 (1.31–2.91)	0.001
≥ 80 years (*n* = 120)				
Mode of diagnosis				
Symptom onset	Reference		Reference	
Surveillance	0.61 (0.37–1.01)	0.055	0.67 (0.37–1.19)	0.17
Age (years)	1.13 (1.02–1.25)	0.022	1.02 (0.91–1.13)	0.76
Gender (Male)	0.86 (0.52–1.41)	0.54	1.09 (0.59–2.00)	0.78
ECOG				
0	Reference		Reference	
≥ 1	1.33 (0.78–2.25)	0.29	1.33 (0.74–2.41)	0.34
CKD (Yes)	1.03 (0.62–1.72)	0.90	1.11 (0.61–2.02)	0.74
Etiology				
Viral	Reference		Reference	
Non-viral	1.14 (0.69–1.88)	0.61	1.33 (0.77–2.32)	0.31
Initial treatment (Yes)	0.20 (0.11–0.37)	< 0.001	0.25 (0.13–0.49)	< 0.001
ALBI grade				
1	Reference		Reference	
2–3	3.06 (1.85–5.06)	< 0.001	2.93 (1.68–5.11)	< 0.001
α-fetoprotein (log10 ug/mL)	1.26 (1.06–1.49)	0.008	1.15 (0.94–1.40)	0.18
mUICC stage				
I–II	Reference		Reference	
III–IV	2.79 (1.67–4.66)	< 0.001	2.03 (1.11–3.70)	0.021

HR, hazard ratio; aHR, adjusted hazard ratio; ECOG, Eastern Cooperative Oncology Group; CKD, chronic kidney disease; HCC, hepatocellular carcinoma; ALBI, albumin-bilirubin; AFP, α-fetoprotein; mUICC, modified Union for International Cancer Control.

Model 3: adjusted for age (years), gender (male vs. female), ECOG (0 vs. 1–4), CKD (≥ 60 vs. < 60 mL/min/1.73 m^2^), etiology of HCC (viral vs. non-viral), initial treatment (yes vs. no), ALBI grade (1 vs. 2–3), serum AFP (/log_10_ ug/mL), and mUICC stage (1–2 vs. 3–4).

^a^*p*-values for interaction between diagnosis mode and age were > 0.05.

### Propensity score-matched cohort analysis

**Figure 4:**
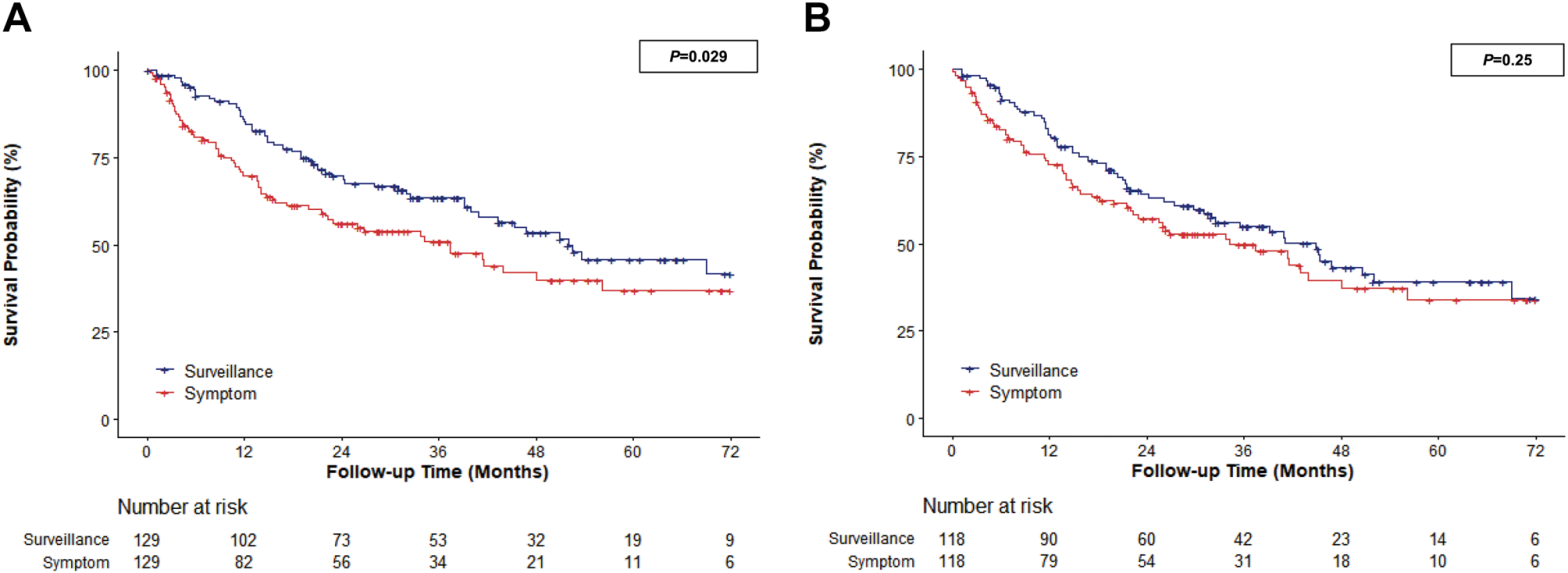
Kaplan–Meier estimates of survival in elderly patients according to diagnosis mode in matched group (over 75 years old) (**A**) propensity score matched cohort that did not include mUICC stage, (**B**) propensity score matched cohort included mUICC stage.

The comparison of baseline characteristics is summarized in Supplementary Table 1. Except for the stage of the first cohort, which did not match the stage variable, there was no differences in clinical or disease-related features between the two matched cohorts. In a propensity score-matched cohort that did not include the mUICC stage, the HR for mortality was 0.67 (95% CI 0.47–0.96) for the surveillance group compared to the symptomatic group (Fig. 4A). When the propensity score-matched cohort included the mUICC stage, the HR for mortality was 0.81 (95% CI 0.57–1.16) for the surveillance group compared to the symptomatic group (Fig. 4B).

## Discussion

In this study, we observed an association between the diagnosis mode and OS in HCC patients aged 75 years or older. Those diagnosed with HCC through surveillance showed longer OS than those with a symptom-driven diagnosis. Many baseline characteristics differed between surveillance and symptomatic groups of patients with HCC. Of note, early-stage diagnosis was much more frequent in the surveillance group (mUICC stage I/II: 72.3% vs. 39.1%, *p* < 0.05). The association between OS and diagnosis mode remained significant after adjusting for several potential confounders and mediators that did not include the tumor stage (Table 2, adjusted model 1 and adjusted model 2). However, when the tumor stage was additionally to adjusted, the association between OS and diagnosis mode was no longer statistically significant. The findings were the same in subgroup analysis stratified by age group (75 – 79 years; ≥ 80 years). In propensity score-matched analysis, the surveillance group showed better OS than the symptomatic group when the PS-matched variables did not include the tumor stage. However, the diagnosis mode was not an independent survival factor when the PS-matched variables included the tumor stage. These findings suggest that differences in survival between the surveillance and symptomatic groups can be largely explained by an early-stage diagnosis.

We fitted 3 models with progressive degrees of adjustment for potential confounders to identify associations between surveillance and survival gains in elderly patients beyond the potential confounders and to identify potential confounding factors. The association between reductions in mortality and surveillance was attenuated after adjusting for the HCC stage, suggesting that the tumor stage mediated the association between surveillance and mortality. PS matching was performed by excluding the mUICC stage among the characteristics that differed between the two groups of the entire cohort to reconfirm whether early detection through surveillance played a role in lowering mortality. The mUICC stage was then additionally included. The results were similar to those of the entire cohort. These findings suggest that the difference in the survival between the surveillance and symptomatic groups could be explained by an early tumor stage at diagnosis. Patients diagnosed in the early stage can receive curative treatment and have an expected median survival of more than 5 years, whereas patients diagnosed with advanced HCC can only receive palliative care and have an expected median survival of 2 years^16^. As HCC is usually asymptomatic until progression, early diagnosis can only be achieved through HCC screening. Even among elderly patients, surveillance could improve early detection, which could enhance OS.

HCC guidelines recommend that surveillance should target those who would be potentially eligible for curative treatment that could improve survival^9^. Although surgical resection has been regarded as the principal curative HCC treatment for many years, elderly patients have been considered unsuitable for surgery due to the increased frequency of comorbidities. However, developments in surgical procedures and postoperative care have made surgical resection safe and effective in elderly patients. A previous study has demonstrated no differences in postoperative survival rates or postoperative complications between young and elderly patients, even those with many comorbidities^17^. Furthermore, thermal ablation (radiofrequency or microwave ablation) is recommended for curative therapy in patients with early-stage HCC who are ineligible for surgery or refuse surgery^9^. Radiofrequency ablation and surgical treatment had comparable patient survival rates in a randomized controlled study^18^. Several studies have reported the efficacy and safety of radiofrequency ablation in elderly patients^19,20^. Beyond surgery or thermal ablation, results from recently conducted trials have demonstrated that transarterial radio-embolization has curative outcomes for early HCC^21,22^. Various curative-intent loco-regional therapies have been developed and introduced to treat HCC^23^. Age is no longer an impediment to receiving curative therapy. Considering that the early detection of cancer through surveillance can lead to adequate curative treatment even in older patients, it is worthwhile to adopt surveillance for elderly patients.

The ultimate goal of surveillance is to obtain a survival benefit. Clinical guidelines recommend incorporating life expectancy into cancer screening decisions. Surveillance is recommended when life expectancy is more than 10 years^24,25^. According to life expectancy data for 2021 from the Centers for Disease Control and Prevention (CDC) in the United States, at the age of 75, all races have an average life expectancy of 11.5 years (10.6 years for men and 11.3 years for women) and 8.6 years at the age of 80 (www.cdc.gov). In South Korea, 2021 national statistics revealed a life expectancy of 13.4 years for 75-year-old adults and 9.9 years for 80-year-old adults (www.kosis.kr). Life expectancy, together with better outcomes in the surveillance group in this study, suggests that HCC surveillance may benefit elderly patients over the age of 75 and even over 80.

This study also had some limitations. This observational study showed an association between better survival in the surveillance group than in the symptomatic group by analyzing newly diagnosed elderly HCC patients. As we only assessed patients diagnosed with HCC, we were unable to prospectively assess the proportion of patients who participated in surveillance according to guidelines. In the similar context, since this is a retrospective research, the overall risk and benefit of a surveillance program in elderly patients could not be answered by this study and require further evaluation. In particular, because our study is a retrospective one of individuals diagnosed with HCC, we were unable to assess the cost aspects (psychological, financial, and medical) of patients who underwent surveillance but were not diagnosed with it. The physical, financial, or psychological harms related to the overdiagnosis or overtreatment of screening elderly patients should be extensively assessed through prospective randomized controlled studies before applying HCC surveillance programs to this population. However, this study sufficiently showed the value of surveillance because it greatly contributed to improvements in the survival rates of elderly patients. Moreover, since the surveillance is conducted on regularly with the purpose of obtaining a diagnosis before symptoms manifest, our study excluded patients who were identified accidently or in an unknown, but on the other hand, it may have caused selection bias. In addition, although we showed that HCC surveillance could be considered for patients over 80 years, supported by better outcomes in the surveillance group compared to the symptomatic group of elderly patients over 80, the size of the studied population was relatively small. In addition, the cut-off age at which surveillance translates to better outcomes could not be assessed. Life expectancy differs by ethnicity and region. Hence, the findings of this study may not be generalizable to areas where life expectancies are different.

In conclusion, we observed better survival in the surveillance group than in the symptomatic group of elderly patients aged 75 years or older who were newly diagnosed HCC. This better survival was largely mediated by earlier tumor stage at diagnosis. This suggests that, even in patients aged 75 years or older, HCC surveillance has shown a significant advantage in terms of improving survival rates, hence active surveillance should be undertaken even in this age group. Although future randomized controlled trials are required to assess more accurate overall benefits and risks, active HCC surveillance in elderly individuals may need to be continued, considering their life expectancy and survival benefits of HCC surveillance.

### Supplementary Information

Supplementary Table 1.

## Data Availability

The data underlying this article cannot be shared publicly, given the privacy of the individuals who participated in the study. The data will be shared upon reasonable request to the corresponding author.
