# Supplementary material for: Hepatocellular carcinoma outcomes and potential implications for surveillance in elderly patients
Source: Sci Rep. 2024 Jul 4;14:15418. doi: 10.1038/s41598-024-66253-0 (PMC11224371; doi:10.1038/s41598-024-66253-0)
Supplement: Supplementary file 1 — Supplementary Table 1. [file 41598_2024_66253_MOESM1_ESM.docx]

**Supplement Table 1.** Baseline characteristics of individuals in propensity-matched cohorts.

|  | **Matched cohort 1** | | | | **Matched cohort 2** | | | |
| --- | --- | --- | --- | --- | --- | --- | --- | --- |
|  | **Matched surveillance (*n* = 129)** | **Matched Sx  (*n* = 129)** | ***p*-value** | **SMD** | **Matched surveillance (*n* = 118)** | **Matched Sx (*n* = 118)** | ***p*-value** | **SMD** |
| **Age (years)** |  |  | 0.89 | - |  |  | 1.00 | - |
| 75-80 | 88 (68.2%) | 89 (69.0%) |  | -0.017 | 84 (71.2%) | 84 (71.2%) |  | 0.000 |
| ≥80 | 41 (31.8%) | 40 (31.0%) |  | 0.017 | 34 (28.8%) | 34 (28.8%) |  | 0.000 |
| **Gender (Male)** | 82 (63.6%) | 90 (69.8%) | 0.29 | -0.132 | 77 (65.3%) | 78 (66.1%) | 0.89 | -0.018 |
| **ECOG** |  |  | 0.89 | - |  |  | 0.67 | - |
| 0 | 92 (71.3%) | 91 (70.5%) |  | 0.017 | 83 (70.3%) | 80 (67.8%) |  | 0.055 |
| ≥ 1 | 37 (28.7%) | 38 (29.5%) |  | -0.017 | 35 (29.7%) | 38 (32.2%) |  | -0.055 |
| **CKD** | 47 (36.4%) | 43 (33.3%) | 0.60 | 0.065 | 40 (33.9%) | 41 (34.8%) | 0.89 | -0.018 |
| **Etiology** |  |  | 0.61 | - |  |  | 0.69 | - |
| Viral | 54 (41.9%) | 50 (38.8%) |  | 0.063 | 53 (44.9%) | 50 (42.4%) |  | 0.051 |
| Non-viral | 75 (58.1%) | 79 (61.2%) |  | -0.063 | 65 (55.1%) | 68 (57.6%) |  | -0.051 |
| **ALBI grade** |  |  | 0.80 | - |  |  | 0.90 | - |
| 1 | 74 (57.4%) | 76 (58.9%) |  | -0.031 | 67 (56.8%) | 66 (55.9%) |  | 0.017 |
| 2-3 | 55 (42.6%) | 53 (41.1%) |  | 0.031 | 51 (43.2%) | 52 (44.1%) |  | -0.017 |
| **mUICC stage** |  |  | <0.001 | - |  |  | 0.79 | - |
| I-II | 96 (74.4%) | 57 (44.2%) |  | 0.65 | 66 (55.9%) | 64 (54.2%) |  | 0.034 |
| III-IV | 33 (25.6%) | 72 (55.8%) |  | -0.65 | 52 (44.1%) | 54 (45.8%) |  | -0.034 |
| **Treatment status** (Yes) | 117 (90.7%) | 118 (91.5%) | 0.83 | -0.027 | 107 (90.9%) | 106 (89.8%) | 0.83 | 0.029 |
| **α -Fetoprotein** (/log10 ug/ml) | 1.0 (0.6-1.8) | 1.0 (0.5-1.9) | 0.96 | 0.033 | 1.1 (0.6-2.2) | 1.1 (0.6-2.3) | 0.73 | 0.013 |

**Matched cohort 1**: matched for age (75-80 vs. ≥ 80 years), gender (male vs. female), ECOG (0 vs. 1-4), CKD (≥60 vs. < 60 mL/min/1.73 m^2^), etiology of HCC (viral vs. non-viral), initial treatment (yes vs. no), ALBI grade (1 vs. 2-3), and serum AFP (/log_10_ ug/mL)

**Matched cohort 2:** matched for age (75-80 vs. ≥ 80 years), gender (male vs. female), ECOG (0 vs. 1-4), CKD (≥60 vs. < 60 mL/min/1.73 m^2^), etiology of HCC (viral vs. non-viral), initial treatment (yes vs. no), ALBI grade (1 vs. 2-3), serum AFP (/log_10_ ug/mL), and mUICC stage (1-2 vs. 3-4).

Sx, symptom; SMD, standardized mean difference; ECOG, Eastern Cooperative Oncology Group; CKD, chronic kidney disease; ALBI, albumin-bilirubin; mUICC, modified Union for International Cancer Control; HCC, hepatocellular carcinoma; AFP, alpha-fetoprotein.
